# Recombinant Bivalent Live Vectored Vaccine Against Classical Swine Fever and HP-PRRS Revealed Adequate Heterogeneous Protection Against NADC30-Like Strain

**DOI:** 10.3389/fmicb.2021.822749

**Published:** 2022-01-05

**Authors:** Liwei Li, Jinxia Chen, Zhengda Cao, Yunlei Cao, Ziqiang Guo, Wu Tong, Yanjun Zhou, Guoxin Li, Yifeng Jiang, Changlong Liu, Lingxue Yu, Sina Qiao, Jiachen Liu, Guangzhi Tong, Fei Gao

**Affiliations:** ^1^Shanghai Veterinary Research Institute, Chinese Academy of Agricultural Sciences, Shanghai, China; ^2^Jiangsu Co-innovation Center for the Prevention and Control of Important Animal Infectious Disease and Zoonoses, Yangzhou University, Yangzhou, China

**Keywords:** rPRRSV-E2, live vectored vaccine, NADC30-like strain, heterologous protection, challenge model

## Abstract

The recombinant bivalent live vectored vaccine rPRRSV-E2 has been proved to be a favorable genetic engineering vaccine against classical swine fever (CSF) and highly pathogenic porcine reproductive and respiratory syndrome (HP-PRRS). NADC30-like strains have recently emerged in China and caused severe disease, and it is necessary to evaluate the vaccine candidate for the currently circulating viruses. This study established a good challenge model to evaluate the candidate rPRRSV-E2 vaccine in preventing infection with a representative NADC30-like strain (ZJqz21). It was shown that the challenge control piglets displayed clinical signs typical of PRRSV, including a persistent fever, dyspnea, moderate interstitial pneumonia, lymph node congestion, and viremia. In contrast, the rPRRSV-E2 vaccination significantly alleviated the clinical signs, yielded a high level of antibodies, provided adequate protection against challenge with ZJqz21, and inhibited viral shedding and the viral load in target tissues. Our results demonstrated that the recombinant bivalent live vectored vaccine strain rPRRSV-E2 can provide efficient protection against the challenge of heterologous circulating NADC30-like strain and could be a promising vaccine candidate for the swine industry.

## Introduction

Porcine reproductive and respiratory syndrome virus (PRRSV) is the causal agent of the porcine reproductive and respiratory syndrome (PRRS) and is one of the most economically infectious illnesses of the swine industry worldwide. PRRSV belongs to the order *Nidovirales*, family *Arteriviridae* and can be classified into PRRSV-1 and PRRSV-2 with Lelystad and VR-2332 as representative strains (Cavanagh, 1997; Lunney et al., 2016). These two genotypes share approximately 60% nucleotide identity (Darwich et al., 2011).

PRRSV was first reported in 1996 in China. Over the past 20 years, PRRSV has been the cause of major diseases in the swine industry, and PRRSV-2 strains are highly predominant within the field (An et al., 2007). After 2006, highly pathogenic PRRSV (HP-RRRSV) became the predominantly epidemic strain on the farms (Tian et al., 2007, 2009; Zhou et al., 2008). Since 2013, PRRSV infection during routine clinical detection has significantly increased. The recurrent PRRS pandemic was attributed to the emergence of NADC30-like strains, which were likely imported from North America and rapidly spread widely throughout China (Zhao et al., 2015; Zhou et al., 2015; Li et al., 2017). The NADC30-like and HP-PRRSV-like strains currently circulate on pig farms and have high clinical detection rates (Li et al., 2016; Guo et al., 2019).

According to the global PRRSV classification system, the abundance of PRRSV-2 strains in China can be classified into four lineages: NADC30-like (lineage 1), QYYZ-like (lineage 3), VR-2332-like (lineage 5), and JXA1-like/CH-1a-like (lineage 8) lineages (Shi et al., 2010a,b). Extensive genetic variations exist between the strains in each viral subtype, and genetically distinct PRRSV variants display different clinical characteristics and virulence (Guo et al., 2018). rPRRSV-E2 was a recombinant bivalent live vectored vaccine and capable of expressing the classical swine fever virus (CSFV) E2 protein in the backbone of a commercial modified live virus (MLV) vaccine strain, HuN4-F112, which was classified into lineage 8 (Gao et al., 2018). rPRRSV-E2 could provide 100% protection against HP-PRRSV (lineage 8), but it remained to be answered about the protective effect of rPRRSV-E2 for resisting challenge with NADC30-like (lineage 1) strain.

NADC30-like PRRSVs are not as pathogenic as HP-RRRSV but can be distinguished by a greater probability of recombination with other PRRSV strains, leading to different virulence (Zhang et al., 2016). Outbreaks of NADC30-like PRRSV in vaccinated swine herds indicate that the current commercial PRRSV vaccines cannot completely protect against infection with NADC30-like strains (Tian, 2017). This study evaluated the current vaccine candidate rPRRSV-E2 against infection with a heterologous strain and demonstrated that rPRRSV-E2 vaccination exerted superior efficiency at decreasing the clinical signs and viral infection NADC30-like strain in piglets.

## Materials and Methods

### Virus Isolation and Viral Characteristics Analyses

The disease materials were obtained from a pig farm in Zhejiang province, China, in 2020. Three sick piglets displayed high fever, dyspnea, and tachypnea. Lung samples were ground and subjected to three freeze-thaw cycles in RPMI-1640 medium (Gibco, Thermo Fisher Scientific, Waltham, MA). Samples were detected by PRRSV ORF5-specific primers, and the results appeared to be positive (data not shown). (PAMs) were infected with the supernatants for 1 h and subsequently cultured in RPMI-1640 medium containing 10% fetal bovine serum (FBS; Gibco). Daily observations were performed until obvious cytopathic effects (CPEs) appeared. Specific monoclonal antibodies termed SDOW17 (Rural Technologies) were used for an indirect immune fluorescent assay (IFA) to confirm the presence of the PRRSV N protein.

### Complete Genome Sequencing and Phylogenetic Analysis of ZJqz21

The total RNA was extracted from ZJqz21-infected PAMs using TRIzol (Invitrogen, Thermo Fisher Scientific, Waltham, MA). A PrimeScript™ 1st Strand cDNA Synthesis Kit (TaKaRa, Dalian, China) was used for reverse transcription. The complete ZJqz21 genome was amplified by seven overlapping primers using the cDNA template. The sequences of all primers used for genome amplification will be made available upon request. Purified PCR products were sequenced by Personalbio (Shanghai, China). A total of 44 representative PRRSV isolates were collected from the GenBank database (Table 1), and multiple sequence alignments were performed using Clustal X. Phylogenetic trees of the complete genome and ORF5 sequences were constructed by MEGA 6.0 using the neighbor-joining method. Bootstrap confidence values from 1,000 replicates were used to analyze the evolutionary relationship of ZJqz21.

**TABLE 1:** Reference strains in this study.

NO	Isolate	Country	Year	Accession no.
1	Lelystad virus	Europe	1991	M96262.2
2	VR-2332	United States	1992	AY150564.1
3	RespPRRS MLV	United States	1994	AF066183.4
4	CH-1a	China	1996	AY032626.1
5	CH-1R	China	2008	EU807840.1
6	NB/04	China	2004	FJ536165.1
7	JXA1	China	2006	EF112445.1
8	HUN4	China	2007	EF635006.1
9	NADC30	United States	2012	MH500776.1
10	WUH4	China	2012	JQ326271.1
11	HB-1/3.9	China	2007	EU360130.1
12	HB(sh)-1/3.9	China	2002	AY150312.1
13	GDsg	China	2016	KX621003.1
14	GDQY1	China	2011	JN387271.1
15	QYYZ	China	2010	JQ308798.1
16	rJXwn06	China	2009	MF187956.1
17	GM2	China	2012	JN662424.1
18	HN2007	China	2009	EU880437.2
19	SHH	China	2007	EU106888.1
20	CH-YY	China	2019	MK450365.1
21	TP	China	2008	EU864233.1
22	TJ	China	2008	EU860248.1
23	GD	China	2008	EU825724.1
24	IA/2014/NADC34	United States	2017	MF326985.1
25	CH/2018/NCV-Anheal-1	China	2018	MH370474.1
26	FJ0908	China	2018	MK202794.1
27	PRRSV-ZDXYL-China-2018-2	China	2019	MK453050.1
28	HLJZD30-1902	China	2019	MN648055.1
29	RFLP 1-4-4 lineage 1C variant	United States	2021	Mw887655.1
30	A2MC2	United States	2011	JQ087873.1
31	FJLIUY-2017	China	2017	MG011718.1
32	15LN3	China	2016	KX815425.1
33	SDYG1606	China	2016	KY053458.1
34	HH08	China	2012	JX679179.1
35	GD-KP	China	2016	KU978619.1
36	FJFS	China	2015	KP998476.1
37	SD110-1608	China	2019	MK780825.1
38	SDqd1501	China	2019	MN642099.1
39	PRRSV 2	United States	1995	U87392.3
40	SD1612-1	China	2019	MN119304.1
41	GZ106	China	2014	KJ541663.1
42	CH2004	China	2009	EU880439.2
43	CH2002	China	2009	EU880438.2
44	HK13	China	2013	KF287140.1

### Viruses Used for Vaccination and Challenge

PRRSV-permissive MARC-145 cells were cultured and maintained in Minimum Essential Medium Eagle (MEM; Sigma-Aldrich Corporation, CA, United States) with 10% FBS or 2% FBS at 37°C under humidity of 5% CO_2_. The vaccination strain, rPRRSV-E2, and challenge virus strain, ZJqz21, were propagated and obtained when approximately 80% CPE of the MARC-145 cells appeared. The two viruses were titrated and used for vaccination and challenge, respectively. The doses used in the experiments are shown in Table 2.

**TABLE 2:** The experimental design.

Groups (*n* = 5)	Vaccination			Challenge
	Vaccine	Times	Doses	
Vaccinated	rPRRSV-E2	Once	10^5.0^ TCID_50_	ZJqz21 (10^5.0^ TCID_50_)
BC group	DMEM	Once	2 ml	ZJqz21 (10^5.0^ TCID_50_)
Mock	DMEM	Once	2 ml	None

### Evaluation of rPRRSV-E2 Immune Efficacy Against ZJqz21 in Piglets

A professional veterinary pathologist was invited to guide and perform professional animal experimental operations. A total of 15, 30-day-old landrace PRRSV- and CSFV-free piglets were selected for immune efficacy experiments to assess the effect of rPRRSV-E2 against ZJqz21 and divided into three groups. Each group contained five piglets fed separately, as shown in Table 2. A 2 ml dose of rPRRSV-E2 (dosage: 10^5.0^ TCID_50_) was used to vaccinate each piglet in the vaccinated and challenge control piglets (BC groups; PRRSV S/P < 0.4; *n* = 5) and 2 ml of ZJqz21 (dosage: 10^5.0^ TCID_50_) was inoculated at 28 days post-vaccination (dpv). A cervical intramuscular injection was used for vaccination and challenge. Each piglet was injected with 2 ml DMEM in the mock group. General health status and clinical symptoms were monitored daily, and the rectal temperatures were measured daily during the experimental period. PRRS clinical symptoms were recorded and scored as described previously (Halbur et al., 1995; Thanawongnuwech et al., 2000; Li et al., 2014). All surviving piglets were euthanized and autopsied 21 days post-infection (dpi). The sera were collected every 7 days after rPRRSV-E2 vaccination. After ZJqz21 inoculation, sera were collected every 2 or 3 days. Real-time RT-qPCR evaluated the level of viral RNA replication in the serum samples containing PRRSV viremia and virus shedding and viral load in the tissues (Jiang et al., 2015; Gao et al., 2018, 2020b). An evaluation of lung lesions in each of the three groups was performed as previously described (Jiang et al., 2015; Gao et al., 2018, 2020a). For pathological observations, a pathological section analysis and detection of the viral load in the tissues of piglets infected with the ZJqz21 strain was performed. The heart, lung, inguinal lymph nodes, mesenteric lymph nodes, mandibular lymph nodes, spleen, kidneys, and tonsils were collected from each piglet.

### Detection of Specific Humoral Immunity After Vaccination and Challenge

The PRRSV- and CSFV-specific antibody titers in the serum samples collected at the specified time points were tested with the commercial ELISA kits (IDEXX Laboratories, Westbrook, No. 06-40959-04 and No. 06-43230-06) as previously described (Gao et al., 2018). Based on these values, the level of humoral immunity was displayed by drawing curves with GraphPad Prism 6.0.

### Determination of the Viral Load in Tissues

Tissue samples from the heart, lung, inguinal lymph nodes, mesenteric lymph nodes, mandibular lymph nodes, spleen, kidneys, and tonsils were collected by multi-point sample collection. A certain amount of each tissue sample was gathered, 1 ml of sterile PBS was added for tissue homogenates, and grinding treated tissue homogenates were centrifuged at 4°C (9,500 × g for 10 min), and the supernatant was cryopreserved in a 1.5 ml centrifuge tube. The RNA of 200 μL-treated homogenate supernatant was extracted using the QIAcube HT nucleic acid autotractor according to the QIAcube HT Plasticware kit instructions. RT-qPCR was performed, and Shanghai Sunny Biotechnology Co., Ltd., synthesized the primer pairs and probes.

### Statistical Analysis of Experimental Data

A Student′s *t*-test was used for statistical differences analysis in the study. SPSS 14.0 and GraphPad Prism software (version 6.0) was used to analyze and chart the clinical symptom scores, lung lesion scores, viral shedding, and viral load values. A *P*-value < 0.05 was considered to be statistically significant.

## Results

### ZJqz21 Was Identified as a Heterologous Strain to rPRRSV-E2

**FIGURE 1:**
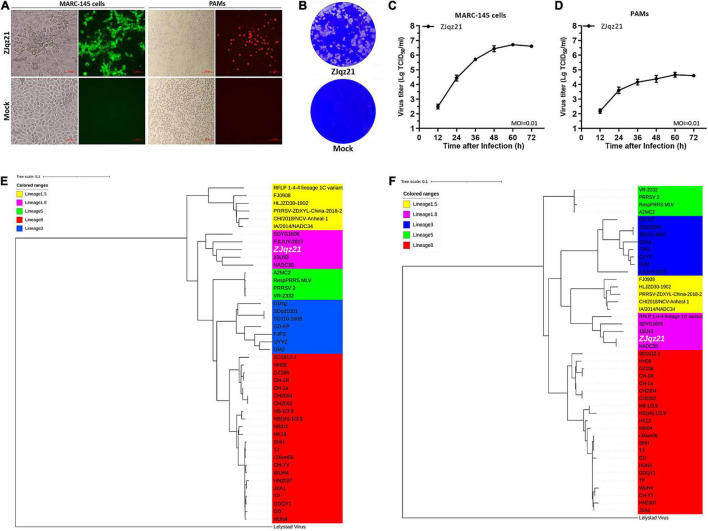
**(A)** Cytopathic effects (CPE) and immunofluorescence staining against the N protein in Primary porcine alveolar macrophages (PAMs) and MARC-145 cells at 36 hpi with ZJqz21 (MOI = 0.1). Scale bar = 100 μm. **(B)** Plaque morphology. MARC-145 cells were infected with ZJqz21 (MOI = 0.001). The mock control represented non-infected MARC-145 cells. The MARC-145 cells were stained with crystal violet at 5 days post-infection. Virus growth kinetics in MARC-145 cells **(C)** and PAMs **(D)**. Data represent the mean ± standard deviation (SD) of three independent experiments. Phylogenetic trees of ZJqz21 based on the complete genome **(E)** and GP5 **(F)** nucleotide sequences. A white color highlighted the ZJqz21 strain. The scale bar indicated the number of nucleotide substitutions per site.

To evaluate the immune efficacy of rPRRSV-E2 against the heterologous strain, we first identified a NADC30 epidemic strain. In 2020, ZJqz21 was successfully isolated from PAMs from a RespPRRS MLV-vaccinated piglet in China. PRRSV-specific CPE was observed in the P2 passage, and the existence of PRRSV was further identified by IFA (Figure 1A). As shown in Figure 1A, ZJqz21 could cause severe CPE. An IFA analysis was carried out on MARC-145 cells, or PAMs infected with ZJqz21 (Figure 1A). The viral growth characteristics of ZJqz21 are presented by multi-step growth curves and a plaque morphology analysis (Figures 1B–D).

The complete sequences of the ZJqz21 genome have been uploaded into GenBank (accession no. OK274266). The complete ZJqz21 genome is 15,042 nt in length, including poly(A) tails. The ZJqz21 genome shared 86.9, 87.0, 84.4, 86.8, and 90.1% homology in the nucleotide sequence with CH-1a, JXA1, GM2, VR2332, and NADC30, respectively (Table 3). Each region of the ZJqz21 genome was further compared with those representative strains, and the results showed that ZJqz21 shared the highest identity in each region with the NADC30 strain. Amino acid sequence alignment results revealed that Nsp2 of ZJqz21 has a discontinuous 131-aa (111 + 1 + 19-aa) deletion compared with VR-2332 (data not shown), which are the typical characteristics of the NADC30-like PRRSV. The ORF5 of ZJqz21 displayed higher nucleotide and amino acid identity (94.7%, 93.0%) with NADC30 (Table 3).

**TABLE 3:** Nucleotide and amino acid sequence identity (%) of ZJqz21 as compared to five representative PRRSV strains.

Regions	CH-1a	JXA1	GM2	VR-2332	NADC30

	Pairwise % identity (nt/aa)				
Genome	86.9	87.0	84.4	86.8	90.1
5′UTR	95.3	97.9	93.2	90.0	93.2
ORF1a	80.0/84.0	79.5/85.5	75.5/80.2	80.3/84.6	88.1/88.5
ORF1b	89.9/95.0	90.1/95.8	88.4/95.1	88.9/94.9	89.3/95.3
ORF2a	87.5/88.7	86.3/86.4	86.8/90.3	88.3/90.3	96.1/94.9
ORF2b	91.0/90.5	89.6/93.2	92.3/90.5	91.0/89.2	95.9/93.2
ORF3	83.7/81.2	83.7/80.8	82.7/82.4	84.1/82.4	96.2/95.7
ORF4	87.7/88.3	86.6/88.3	86.8/87.2	88.3/88.3	97.4/97.8
ORF5	87.7/87.6	86.1/86.1	84.7/84.6	86.1/83.6	94.7/93.0
ORF6	88.0/92.0	89.0/93.1	90.5/92.6	90.1/92.6	98.3/99.4
ORF7	92.2/92.7	91.1/91.1	87.1/87.9	93.3/94.4	96.8/98.4
3′UTR	89.4	89.4	88.7	94.0	97.4

Phylogenetic trees were constructed according to the complete genome and ZJqz21 ORF5 sequences. The results demonstrated that PRRSV-2 strains could be divided into the following five lineages: RFLP 1-4-4 (lineage 1.5), NADC30-like (lineage 1.8), VR-2332-like (lineage 5), GM2-like (lineage 3), and JXA1-like/CH-1a-like (lineage 8) based on the complete genome sequence. ZJqz21 was classified into lineage 1.8, represented by NADC30 based on full nucleotide genome and ORF5 sequences (Figures 1E,F). These findings revealed that ZJqz21 belonged to lineage 1.8 and had characteristics of the NADC30 strain, which can be used as the heterologous challenge strain to rPRRSV-E2.

### rPRRSV-E2 Protected the Piglets From the Challenge of the Heterologous NADC30-Like Strain ZJqz21

**FIGURE 2:**
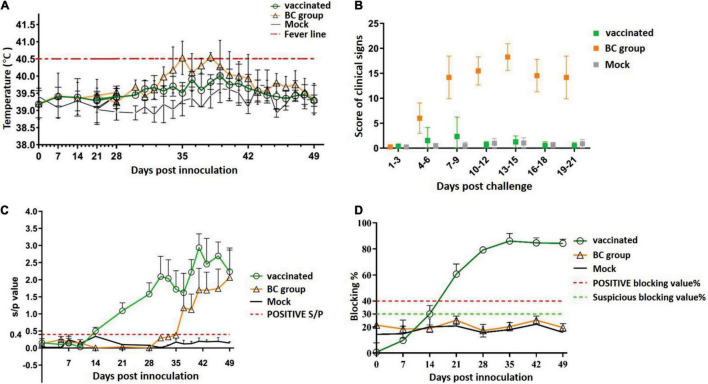
Serological response after rPRRSV-E2 vaccination and scoring of clinical signs following challenge with the novel NADC30-like strain, ZJqz21. **(A)** Daily rectal temperature record and analysis following rPRRSV-E2 vaccination and ZJqz21 challenge. **(B)** Scoring and charting for clinical symptoms following rPRRSV-E2 vaccination and ZJqz21 challenge. Scoring data were indicated as the means ± standard deviations. **(C)** The S/P value assessed the PRRSV-specific humoral immune response (IDEXX PRRS X3) identified from serum samples collected at the indicated time points from piglets in the vaccinated, BC, and mock groups. **(D)** CSFV-specific antibodies in the three groups were tested in the serum samples collected in the experiments using an IDEXX CSFV Ab ELISA kit.

Next, the efficacy of rPRRSV-E2 against the heterologous strain ZJqz21 in piglets was tested. The BC group and vaccinated piglets (vaccinated group) were challenged with the ZJqz21 strain. In the BC group, the body temperature rose at 3 days post-challenge (dpc) and reached the peak temperature at about 11 dpc. Among them, three of the pigs reached 41.2°C. The rectal temperatures of the piglets in the mock group were below 40.5°C throughout the experimental period. In the vaccinated group, one piglet developed symptoms of fever, which lasted from 8 to 12 dpc, before returning to normal. The rectal temperature of the other pigs in this group was regular (Figure 2A). Piglets in the BC group showed typical PRRS symptoms (e.g., high fever, cough, loss of appetite, lethargy, and frequent paralysis) from 4 dpc (Figure 2B). The clinical sign scores in the BC group from 8 to 21 dpc were significantly higher than those in the vaccinated and mock groups (*P* < 0.001, Figure 2B). Of note, all ZJqz21-infected piglets survived throughout the study. The ZJqz21 strain was found to be a moderately virulent isolate. Compared with piglets in the BC group, the piglets vaccinated with rPRRSV-E2 were challenged with the ZJqz21 strain, and there were almost no signs of a high fever or other abnormal clinical symptoms. Only one pig in the vaccinated group developed symptoms of clinical fever, which lasted for 5 days. The rectal temperature was approximately 41°C; however, the mental state was normal and did not show serious clinical symptoms (Figure 2A).

The antibody reaction induced by rPRRSV-E2 and the subsequent challenge by ZJqz21 were examined to determine the protective efficacy of rPRRSV-E2 against the heterologous strain ZJqz21. Based on the serum collected at 0, 7, 14, 21, 28, 35, 42, and 49 dpi, the immune response to PRRSV and CSFV was analyzed. The group inoculated with rPRRSV-E2 produced PRRSV-specific immune effects. All of the piglets vaccinated with rPRRSV-E2 showed seroconversion by 14 dpv, and the average S/P showed a peak of 1.75 ± 0.39 at 28 dpv (Figure 2C), demonstrating that rPRRSV-E2 vaccination-induced high-level PRRSV-specific antibodies. At the same time, the level of CSFV-specific antibodies in the piglets inoculated with rPRRSV-E2 began to increase at 7 dpv and rapidly climbed to the average peak titer at 35 dpv. At about 2 weeks after vaccination with the rPRRSV-E2 vaccine, CSFV-specific antibodies in piglets were seroconverted in the vaccination group and reached 82.6 ± 2.1% after 28 dpv (Figure 2D). Following the viral challenge, the S/P values indicated that the PRRSV-specific antibodies in the BC group increased from 10 dpc and were higher than those of the mock group (Figure 2C). During the study period, PRRSV-specific and CSFV-specific antibodies in the piglets of the mock group remained negative (Figures 2C,D).

### rPRRSV-E2 Vaccination Alleviated the Clinical Signs Typical of PRRSV Caused by ZJqz21

**FIGURE 3:**
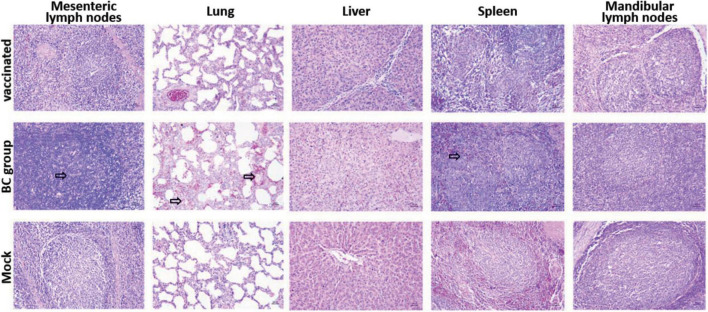
Histopathology analysis by hematoxylin-eosin staining in three groups. Each group’s microscopic figures of the lung, liver, spleen, mesenteric lymph nodes, and mandibular lymph nodes are shown and observed at a magnification of × 200. The infiltration of inflammatory cells around the alveolar septa and alveolar spaces was shown clearly by black arrows.

All piglets in rPRRSV-E2 vaccinated, and mock groups were generally normal and did not show obvious pathological damage typical of PRRS disease. Piglets inoculated with the ZJqz21 strain (BC group) showed severe pathological changes, including tonsil necrosis and hemorrhage, congestion, and swelling of the lymph nodes, bladder, and petechial kidney hemorrhage. Table 4 describes the overall pathological changes in the three groups. Typical pathological signs of PRRSV were observed in the BC group, such as lung lesions, enlarged bleeding of the mesenteric and inguinal lymph nodes, hemorrhage and necrosis of the spleen, and hemorrhaging in the throat and kidney, and tonsil (Table 4). An analysis of the macroscopic score in the lungs showed that infected piglets in the BC group showed severe pathological changes in the lung, significantly higher than those in the inoculation and mock groups (Table 5). The BC group displayed typical interstitial pneumonia and obvious microscopic lung lesions, including exfoliated epithelial cells in the bronchioles and collapsed alveoli infiltrating numerous inflammatory cells in alveolar spaces (Figure 3). The lymph nodes of piglets inoculated with ZJqz21 showed lower proliferation of cortical lymphocytes and enlargement and hyperplasia of the lymphatic nodules (Figure 3). During the entire study period, neither macroscopic nor histological changes in the lung and lymph node lesions were observed in the vaccinated piglets and negative control piglets (Figure 3), indicating that rPRRSV-E2 vaccination significantly alleviated the clinical signs caused by ZJqz21.

**TABLE 4:** Gross evaluation and pathological examination of piglets in three groups.

	Group no. (n)		Vaccinated (*n* = 5)	BC group (*n* = 5)	Mock (*n* = 5)
Gross evaluation	Lung	Hemorrhage	1/5	5/5	1/5
and pathological		Lesion	1/5	4/5	0/5
examination of	Inguinal lymph node	Hemorrhage	0/5	4/5	1/5
piglets infected		Swelling	1/5	5/5	1/5
with ZJqz21	Mesenteric lymph node	Hemorrhage	0/5	4/5	1/5
		Swelling	1/5	5/5	1/5
	Spleen	Hemorrhage	0/5	3/5	0/5
		Necrosis	0/5	2/5	0/5
	Kidney	Blood spots	0/5	1/5	0/5
	Tonsil	Hemorrhage	1/5	4/5	0/5
	Brain	Hemorrhage	0/5	2/5	0/5
	Throats	Hemorrhage	1/5	4/5	1/5
	Thoracic adhesion		0/5	3/5	0/5
	Pleural effusion		1/5	2/5	1/5
	Peritoneal adhesion		0/5	1/5	0/5

*Mean values ± SD of macroscopic lung scores ≥ 50 were defined as serious lesions.*

**TABLE 5:** Mean value ± SD of macroscopic scores of the gross lung lesion in three groups.

Designation	Number	Macroscopic score (lung)			
		Mean ± SD	Pathological changes		
			*	**	***
Vaccinated	5	26.30 ± 6.01	4	1	0
BC group	5	68.20 ± 8.98	0	0	5
Mock	5	19.00 ± 3.20	5	0	0

*Macroscopic scores ≤ 30.

**Macroscopic scores > 30 and < 50.

***Macroscopic scores ≥ 50.

### rPRRSV-E2 Vaccination Decreased Viral Replication and Virus Shedding of ZJqz21 in Piglets

**FIGURE 4:**
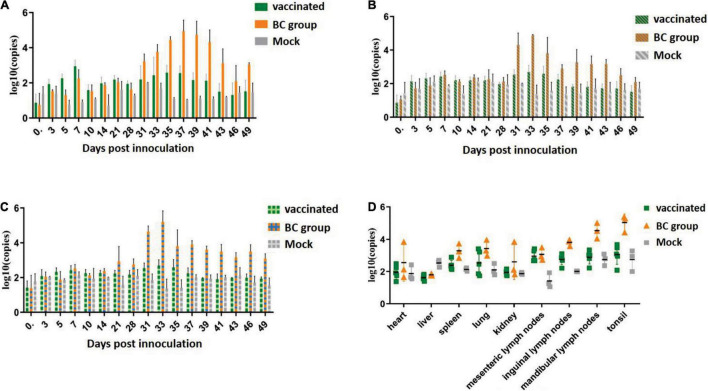
Detection of viremia, virus shedding, and viral load in the tissues by RT-qPCR. **(A)** Viremia detection in experiments assessing the immune efficacy of rPRRSV-E2 against ZJqz21. **(B)** Identification of viral RNA in nasal swabs to assess viral shedding. **(C)** Viral RNA detection in anal swabs to assess viral shedding. **(D)** Detection of the viral load in the lungs, heart, spleen, liver, kidneys, mesenteric lymph nodes, inguinal lymph nodes, mandibular lymph nodes, and tonsils.

RT-qPCR was used to monitor the viral titer in the sera and excreta (including nasal and anal swab samples) and to monitor and evaluate the viral load in tissues and organs. Viremia was detected in all three groups. In the BC group, all piglets inoculated with the ZJqz21 strain showed viremia, which started from 3 dpc and continued to 21 dpc. One of five vaccinated piglets in the vaccinated group exhibited PRRSV-viremia, which started from 3 dpc and lasted for 5 days. Viremia was lower than the negative line at 10 dpc and decreased to an undetectable level until the end of the experiment (Figure 4A), indicating that rPRRSV-E2 vaccination mitigated the viremia caused by ZJqz21. During the entire experiment, it was determined that the Ct value related to virus shedding was close to the detection limit for the nasal and anal samples from the vaccinated group. The level of virus shedding identified in both the nasal and anal swabs of the BC group was high (Figures 4B,C). The numerical values in the viral load of the tissues and organs exhibited similar trends with the detection of virus shedding (Figure 4D). The viral load was extremely high in the mesenteric, inguinal, mandibular lymph nodes, and tonsils of the BC group, indicating that rPRRSV-E2 vaccination also inhibited virus shedding of ZJqz21.

## Discussion

PRRS is an important swine disease rapidly becoming prevalent worldwide and has caused huge economic losses to the pig industry since the 1990s (Lunney et al., 2010). In 2006, there was an HP-PRRSV outbreak in China, posing a serious threat to the Chinese pig industry (Tian et al., 2007, 2009; Zhou et al., 2008). Subsequently, HP-PRRSV with a 30-aa discontinuous deletion in the Nsp2-coding region gradually emerged as the major epidemic strain in China (Zhou et al., 2009, 2014). The emergence of the new lineage 3 (QYYZ-like) and lineage 1 (NADC30-like) viruses in mainland China in 2010 and 2013, respectively, complicated the epidemic situation of PRRS (Lu et al., 2015; Zhao et al., 2015; Zhou et al., 2015). The diversity of PRRSV strains poses a substantial challenge to protecting existing live vaccines. The NADC30-like strain, which is easy to recombine with other PRRSV strains, has become more complex and challenging on pig farms since it became popular in China but shows moderate or relatively low clinical virulence. Since its first outbreak, researchers in China have paid close attention to NADC30-like PRRSV.

Seven commercial MLV vaccines are currently used to control and prevent PRRSV infection in China with their corresponding virus strains, including Ingelvac PRRS MLV/RespPRRS MLV (VR-2332), CH-1R (parental strain CH-1a), HuN4-F112 (HuN4), JXA1-P80 (JXA1), R98 (R98), TJM-F92 (TJ), and GDr180 (GD). These PRRS MLV vaccines are obtained from the classical or HP-PRRSV strains, which can provide adequate protection against genetically homologous strains (Wang et al., 2021); however, the protective effect of MLV vaccines for resisting challenge with heterologous strains is far from conclusive. Zhou et al. (2017) found that the cross-protective efficacy of three different attenuated vaccine strains (including an HP-PRRSV attenuated vaccine strain) against the NADC30-like strain was extremely limited against infection with the NADC30-like virus. Among these strains, Ingelvac PRRS MLV was a commercial vaccine based on the classical PRRSV-2 strain that appeared to exert superior efficiency at reducing clinical fever (Zhou et al., 2017). As a recombinant bivalent live vectored vaccine, rPRRSV-E2 was constructed from the PRRS vaccine virus HuN4-F112, providing 100% protection against HP-PRRSV and CSFV (Tian et al., 2009; Gao et al., 2018). This study demonstrated that rPRRSV-E2 could resist the heterologous NADC30-like strain ZJqz21 infection. Vaccination with rPRRSV-E2 provided good piglet protection and decreased viral replication and virus shedding against heterologous infection with PRRSV strain ZJqz21 (Figures 2–4).

NADC30-like PRRSVs are epidemic in numerous provinces in China. The current commercial vaccines show limited protection in infected piglets. The extensive recombination phenomenon among NADC30-like PRRSVs is a distinctive characteristic of PRRSV (Wang et al., 2018; Zhou et al., 2018; Han et al., 2020). In dealing with complex epidemic situations, there is an urgent need to establish a good challenge model to evaluate the current vaccine candidates in preventing infection with a heterologous strain. In this study, we isolated a novel PRRSV strain, ZJqz21, and identified its genomic characterization, pathogenicity, and application as a challenge model for the heterologous protection of rPRRSV-E2. ZJqz21 was identified and clustered into the NADC30-like lineage (Figure 1). High genetic diversity is a significant characteristic of PPRSV. A previous study constructed the global classification system of PRRSV based on a comprehensive analysis of the complete ORF5 gene sequence. PRRSV-1 was divided into three subtypes (subtypes 1-3) according to the classification system, and PRRSV-2 was classified into 9 lineages with several sublineages in each lineage (Shi et al., 2010b; Zhou et al., 2014). ZJqz21 had a discontinuous 131-aa (111 + 1 + 19-aa) deletion in Nsp2 compared to VR-2332, which was identical to NADC30. A phylogenetic analysis of the full genome and ORF5 sequences showed that ZJqz21 was classified as lineage 1.8 with genetic features from NADC30 (Figure 1). ZJqz21 and NADC30 also belonged to lineage 1.8. The structural proteins only presented 5–8% differences between each other in the amino acid sequence (Table 3). NADC30-like PRRSVs have been recognized as moderately pathogenic isolate strains (Tian, 2017). The pathogenicity of ZJqz21 was evaluated to explore whether it could be applied as the challenge model for the heterologous protection of PRRSV candidate vaccines. Our results showed that none of the piglets died upon challenge with ZJqz21; however, all piglets exhibited typical PRRS clinical symptoms, including fever, cough, inappetence, dyspnea, and tachypnea (Tables 4, 5). Microscopic lung lesions and interstitial pneumonia were observed in ZJqz21-infected piglets (Figure 3). The above results demonstrated that the novel ZJqz21 strain exhibited moderate virulence in piglets and could be used as a challenge model to evaluate the current vaccine candidates in preventing infection with NADC30-like strain.

Nevertheless, in this experiment, rPRRSV-E2 could not perfectly prevent the symptoms of clinical fever after a challenge with the ZJqz21 strain. One out of five pigs in the vaccinated group exhibited clinical fever from day 5 dpc, which lasted for 5 days, and then returned to normal. However, the rectal temperature was not particularly high at around 41°C. Although one piglet in the rPRRSV-E2 vaccinated group had fever symptoms, there were no other more serious clinical symptoms, and there were no significant differences in the clinical symptoms and lung lesions between the vaccinated and mock group (Table 4). There were no typical pathological PRRSV-specific changes in histopathologic slides’ results (Figure 3).

Moreover, there were no significant differences in viremia, viral shedding, or viral load between this pig and other pigs in the same group and those in the mock group (Figure 4). This finding may be related to the infection of piglets with other pathogens. Future studies should confirm that our vaccine candidate strain rPRRSV-E2 displays a good cross-protection effect against heterologous strains and is a good choice for clinical prevention and control of NADC30-like strains.

In conclusion, the current vaccine candidate rPRRSV-E2 can provide good protection against the attack of the moderately pathogenic heterologous PRRSV strain ZJqz21 in piglets. rPRRSV-E2 vaccination alleviated the clinical signs, viral replication, and virus shedding of ZJqz21 in piglets. Our research emphasizes the importance of developing a broad-spectrum vaccine to prevent and control further complicated PRRS epidemics in China.

## Data Availability Statement

The datasets presented in this study can be found in online repositories. The names of the repository/repositories and accession number(s) can be found in the article/supplementary material.

## Ethics Statement

All experimental programs involving pigs were carried out in accordance with the Guidelines for the Nursing and Use of Experimental Animals, and approved by the Ethics Committee of Shanghai Veterinary Research Institute, Chinese Academy of Agricultural Sciences (number SV-20201021-Y02).

## Author Contributions

FG and GT conceived and designed the study. LL and JC wrote the main manuscript text and prepared the figures. ZC, YC, ZG, SQ, and JL performed the experiments. WT, YZ, GL, YJ, CL, and LY prepared the manuscript. All authors reviewed the manuscript and participated in the experiments.

## Conflict of Interest

The authors declare that the research was conducted in the absence of any commercial or financial relationships that could be construed as a potential conflict of interest.

## Publisher’s Note

All claims expressed in this article are solely those of the authors and do not necessarily represent those of their affiliated organizations, or those of the publisher, the editors and the reviewers. Any product that may be evaluated in this article, or claim that may be made by its manufacturer, is not guaranteed or endorsed by the publisher.
